# Walking with unilateral ankle-foot unloading: a comparative biomechanical analysis of three assistive devices

**DOI:** 10.1186/s12984-024-01333-w

**Published:** 2024-04-30

**Authors:** Eshraq Saffuri, Eyal Izak, Yinon Tal, Einat Kodesh, Yoram Epstein, Dana Solav

**Affiliations:** 1 Faculty of Mechanical Engineering, Technion Israel Institute of Technology, Haifa, Israel; 2 School of Public Health, Faculty of Medicine, Tel Aviv University, Tel Aviv, Israel; 3 Department of Physical Therapy, University of Haifa, Haifa, Israel

**Keywords:** Ankle-foot orthosis (AFO), Crutches, Gait analysis, Gait rehabilitation, Hands-free crutch, Joint kinematics, knee crutch, Metabolic cost, Non-weight-bearing

## Abstract

**Background:**

Foot and ankle unloading is essential in various clinical contexts, including ulcers, tendon ruptures, and fractures. Choosing the right assistive device is crucial for functionality and recovery. Yet, research on the impact of devices beyond crutches, particularly ankle-foot orthoses (AFOs) designed to unload the ankle and foot, is limited. This study investigates the effects of three types of devices—forearm crutches, knee crutch, and AFO—on biomechanical, metabolic, and subjective parameters during walking with unilateral ankle-foot unloading.

**Methods:**

Twenty healthy participants walked at a self-selected speed in four conditions: unassisted able-bodied gait, and using three unloading devices, namely forearm crutches, iWalk knee crutch, and ZeroG AFO. Comprehensive measurements, including motion capture, force plates, and metabolic system, were used to assess various spatiotemporal, kinematic, kinetic, and metabolic parameters. Additionally, participants provided subjective feedback through questionnaires. The conditions were compared using a within-subject crossover study design with repeated measures ANOVA.

**Results:**

Significant differences were found between the three devices and able-bodied gait. Among the devices, ZeroG exhibited significantly faster walking speed and lower metabolic cost. For the weight-bearing leg, ZeroG exhibited the shortest stance phase, lowest braking forces, and hip and knee angles most similar to normal gait. However, ankle plantarflexion after push-off using ZeroG was most different from normal gait. IWalk and crutches caused significantly larger center-of-mass mediolateral and vertical fluctuations, respectively. Participants rated the ZeroG as the most stable, but more participants complained it caused excessive pressure and pain. Crutches were rated with the highest perceived exertion and lowest comfort, whereas no significant differences between ZeroG and iWalk were found for these parameters.

**Conclusions:**

Significant differences among the devices were identified across all measurements, aligning with previous studies for crutches and iWalk. ZeroG demonstrated favorable performance in most aspects, highlighting the potential of AFOs in enhancing gait rehabilitation when unloading is necessary. However, poor comfort and atypical sound-side ankle kinematics were evident with ZeroG. These findings can assist clinicians in making educated decisions about prescribing ankle-foot unloading devices and guide the design of improved devices that overcome the limitations of existing solutions.

**Supplementary Information:**

The online version contains supplementary material available at 10.1186/s12984-024-01333-w.

## Background

Numerous medical conditions affect the foot and ankle, including diabetic foot ulcers, Charcot neuroarthropathy, Achilles tendon ruptures, foot and ankle fractures and sprains, and surgical procedures such as ankle replacement or fusion. These conditions often require the patients to unload the affected leg for prolonged durations. For example, previous studies have reported periods of approximately 4–8 weeks for ankle fractures [1], up to 24 weeks for Charcot osteoarthropathy [2], and up to 38 weeks for diabetic ulcers [3]. Consequently, ambulatory assistive devices are commonly prescribed to facilitate ambulation while avoiding undesired weight-bearing of the affected leg [4].

**Fig. 1:**
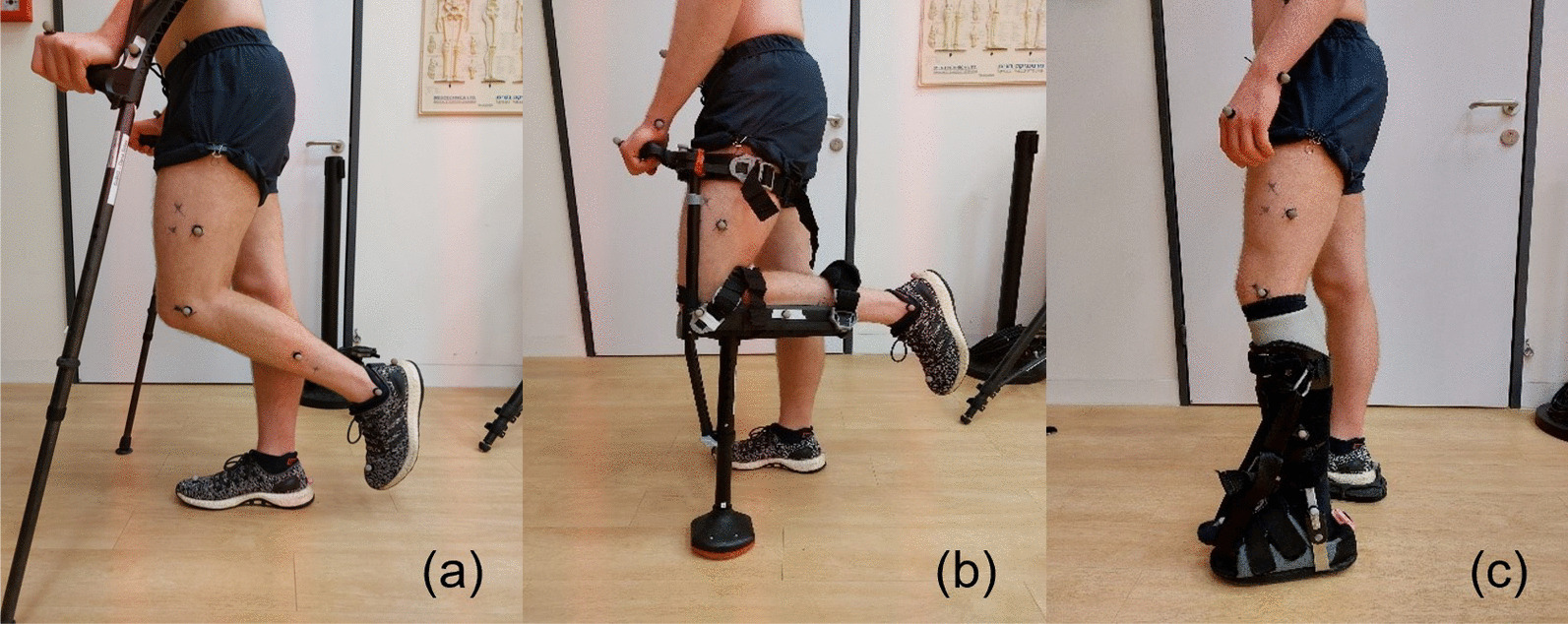
The ankle-foot unloading devices examined in this study: **a** Forearm crutches (CR), **b** iWalk 2.0 (IW), **c** Zero-G Ankle Foot Orthosis (ZG). All the devices were used such that only one foot is weight-bearing and the other one is completely unloaded

Currently, crutches constitute the standard care for enabling patients to walk without loading their ankle or foot [5] (Fig. 1a). Compared to wheelchairs, crutches allow greater mobility and functionality, which are beneficial to patient health and rehabilitation outcomes [6]. However, studies have shown that crutch gait tends to be slower and less energetically efficient than normal gait [5, 7–10], and limits the use of the upper extremities [11]. Compared to normal gait, crutches alter the walking pattern, joint kinematics, and ground reaction force (GRF) patterns [8, 12–14]. The unloading and immobilization of the affected leg may cause muscle atrophy and bone density decrease in the unloaded leg [15–18]. For example, significant reductions in thigh and calf muscle tissue cross-sectional area were found after four weeks of non-weight-bearing in patients with foot fractures [15], and bone density significantly decreased after 6 weeks of non-weight-bearing and continued to decrease even after 6 and 13 weeks of full weight-bearing [18]. Furthermore, crutch usage may lead to increased loading on the weight-bearing leg and upper extremities, which could be detrimental to some patients, particularly in prolonged use [12, 19–22]. Specifically, one-leg swing-through crutch gait has been cautioned against for patients with diseased bones and joints in the lower limb, due to the increased GRFs on the weight-bearing leg [12, 19]. Moreover, the reaction forces transmitted to the arms could be harmful to patients with unsound upper extremities and may be linked to secondary conditions such as hematoma formation, Ulnar nerve compression neuropathy, and Ulnar stress fractures [12, 20–22].

Recently, alternative devices have been proposed for unloading the foot and ankle while walking. One such device is the iWalk knee crutch (iWALKFree, Inc., Long Beach, CA, USA), which enables hands-free gait with a non-weight-bearing status of the lower leg. Its structure consists of a single L-shaped crutch, onto which the user’s shank and thigh are secured via straps. During walking, the knee is maintained at a flexed 90-degree angle, and the foot and ankle are unloaded (Fig. 1b). Previous research has demonstrated that walking with iWalk is associated with reduced upper limb discomfort and superior patient-perceived exertion and preference compared to traditional axillary crutches [23]. Furthermore, a previous study has found that walking with iWalk causes only slight changes in the biomechanical gait patterns examined in the unaffected limb, compared with normal gait [24].

Another type of device that may provide ankle-foot unloading, is an ankle-foot orthosis (AFO). Particularly, an AFO can be designed such that the GRFs are transferred to the shank via a brace tightened around it while maintaining complete unloading of the affected foot. While most AFOs are custom-designed and fitted to patients in specialized clinics, the ZeroG AFO (Certified Orthopedics, Inc., Fort Collins, CO, USA) claims to be the only prefabricated brace that offers complete unloading of the foot and ankle [25] (Fig. 1c). Extensive research exists on AFOs that provide ankle support for conditions such as muscle weakness, motor control deficits, spasticity, and instability [26–29]. Moreover, the effects of braces and casts that provide partial offloading on plantar pressure have been studied [30, 31]. However, to our knowledge, biomechanical analyses of unloading AFOs, such as the ZeroG, have not been published. Nevertheless, we anticipate that unloading AFOs may be advantageous over crutches for several reasons. First, similarly to the knee crutch, they allow for increased mobility of the upper extremities. Second, they allow mobility and loading of the proximal affected leg (above the injured distal part), which may promote a more symmetric and natural walking pattern and lower metabolic cost. Finally, as discussed above, they have the potential to mitigate adverse effects on the proximal bones, joints, and muscles.

This study aims to investigate the biomechanical, metabolic, and subjective outcomes of walking with three different ankle-foot unloading devices compared to unassisted normal gait (NG). Using a within-subject crossover study design with repeated measures, we compared each participant’s NG with their gait using three devices: forearm crutches (CR), iWalk (IW), and ZeroG (ZG), as shown in Fig. 1). The experiments consisted of 20 healthy participants walking at self-selected speed at each of the four conditions. The three-dimensional kinematics of the joints and the center of mass (CoM), the GRFs, and metabolic cost were measured, and the participants provided subjective ratings for stability, perceived exertion, comfort, pressure, and pain through questionnaires. The comparison of joint kinematics and GRF focused on the weight-bearing limb since it allows for direct comparison between the conditions, and because increased GRFs and atypical kinematics of the weight-bearing leg can cause overstrain and secondary injuries, as previous studies have shown in the case of crutches.

We hypothesize that all devices will significantly alter gait parameters compared to normal gait. However, we expect the ZeroG to result in smaller gait alterations because it permits mobility and loading of the unloaded leg’s knee and hip joints. Additionally, we anticipate that crutches would lead to increased GRF peaks and metabolic cost, similar to previous studies, and that iWalk would cause increased CoM mediolateral fluctuations because the locked knee requires hip circumduction to swing the device forward.

The findings from this study could help elucidate the quantitative effects of each device on different biomechanical parameters. This knowledge could be valuable for clinicians in prescribing the most suitable device for each patient’s individual condition, in order to improve their functionality during recovery and minimize the risk of adverse effects associated with the device. This knowledge could be particularly important in cases that require prolonged periods of ankle-foot unloading, as the accumulated impact can become more pronounced. Furthermore, the insights gained from this study could inform the design of improved devices that overcome the limitations of existing devices.

## Methods

### Devices

Three devices for unilateral foot-ankle unloading were selected for this study:

1. Forearm crutches (CR), also known as Canadian crutches. We used the model Access Comfort (FDI FRANCE MÉDICAL, Fitilieu, France), weight: 0.48 kg (Fig. 1a).
2. iWalk (IW), version 2.0 (iWALKFree, Inc., Long Beach, CA, USA), weight: 2.09 kg (Fig. 1b).
3. ZeroG (ZG) AFO (Certified Orthopedics, Inc., Fort Collins, CO, USA), size medium calf lacer and AFO base, weight: 1.49 kg (Fig. 1c). A gel liner ($\hbox {ComfortZone}^{\textrm{TM}}$ Ultra Cushion, Silipos Holding LLC., NY, USA) was worn to add cushioning between the calf lacer and the shank. A shoe leveler (EVENup, Oped Medical, Inc., Buford, GA, USA) was added under the shoe of the weight-bearing foot to equate the length of both legs, as recommended by the manufacturer. During our preliminary testings, we encountered difficulties preventing contact between the forefoot and the AFO base, especially during late stance. To address this issue, we added a wide strap to the calf lacer, which helped provide support to the forefoot and prevent plantarflexion. This ensured that unloading was maintained throughout the gait cycle.

### Study population

Twenty healthy participants were recruited (9 males and 11 females, age: 27.2 ± 5.5 years, height: 167.1 ± 6.9 cm, mass: 65.3 ± 8.9 kg). All participants were free from current injury or any condition that might affect typical walking patterns. Moreover, all participants were within the sizing range suitable for the medium-size ZeroG, per the manufacturer’s fitting instructions. The study was approved by the Institutional Review Board at Technion (#108-2020). Before their inclusion and following a detailed explanation of the study requirements, participants provided written informed consent.

### Experimental protocol

Participants visited the Mechanical Engineering Faculty at the Technion on two consecutive days. On the first visit, a certified physical therapist fitted the devices on the non-dominant leg. Leg dominance was determined by asking participants which leg they would use to kick a ball. Participants were instructed to completely unload their non-dominant leg when walking (i.e., lifting their non-dominant leg completely off the floor using CR, and ensuring the plantar foot surface is unloaded when using ZG). After familiarization with each device, the participants performed a six-minute walking test (6MWT) at a self-selected speed along an indoor 50 m corridor. First, they walked without any device (NG condition) and then with each device in random order. The Oxygen consumption (VO_2_) was measured using a wearable metabolic system (K5, COSMED, Rome, Italy), and the distance walked at each 6MWT was recorded for calculating the mean walking speed. After each condition, the participants were given a ten-minute rest period, during which they filled out a questionnaire, rating their perceived exertion, stability, and comfort, on a 0–10 scale. Additionally, they were asked to indicate any pain or pressure regions caused by the devices (using a body chart) and rate them on a 0–10 scale. On the second visit, the participants arrived at the Technion BRML laboratory, where a 16-camera three-dimensional motion capture system (Vicon Motion Systems Ltd, Oxford, UK) was used to collect kinematic data at 120Hz. Participants were fitted with 39 reflective markers according to the Plug-In-Gait Full body model. Walking trials consisted of walking at a self-selected speed along a 10 m straight walkway equipped with two floor-embedded force plates (OR6-7-1000, AMTI Inc., Watertown, MA, USA), recording the GRF at 960 Hz. For each condition, 10–20 gait cycles (GCs) were recorded, and the conditions were conducted in the same random order as in the first visit.

### Data processing

The metabolic cost for each condition was calculated by normalizing the mean VO_2_ by the participant’s body mass. The data were subsequently normalized by the walking speed (calculated from the walking distance during the 6MWT), which reflects its efficiency, i.e., the aerobic demand per unit of distance walked [32]. The marker trajectories and the GRF data were processed using Nexus 2.9.3 software (Vicon Motion Systems Ltd, Oxford, UK) to extract the hip, knee, and ankle sagittal plane joint angles, body center of mass (CoM) trajectories, and the initial contact (IC) and toe-off (TO) gait events. GCs in which the participant stepped on the edges of the force plate were excluded from the analysis. The raw signals of the joint angles and GRF were filtered using a low-pass Butterworth filter, using a 4th-order filter with a cut-off frequency of 6Hz and a 2nd-order filter with a cut-off frequency of 10 Hz, respectively. For each trial, the GC of the weight-bearing leg was defined between two consecutive ICs, and the stance phase duration was defined from IC to TO. Consequently, all GCs were temporally aligned and interpolated between 0 and 100%. Moreover, the GRFs were normalized by each participant’s body weight. Furthermore, the minimum and maximum local peaks of the joint angles and the anterior-posterior and vertical components of the GRF were identified. Note that the analysis of joint angles and GRF focused on the weight-bearing leg to allow direct comparison between the devices since the unloaded leg is supported differently in each condition (free to move and completely unloaded using CR, loaded from the knee upwards with a fixed knee flexion using IW, and loaded from the shank upwards with the knee free to articulate using ZG).

### Statistical analysis

The statistical analysis was carried out using SAS 9.4 (SAS Institute Inc., Cary, NC). Normality tests were conducted using the Kolmogorov-Smirnoff test for the following parameters: GRF peaks, CoM range of fluctuation, joint angles peaks, walking speed, metabolic cost, stance phase duration, and subjective parameters. To analyze the intra-subject differences, a one-way Analysis of Variance (ANOVA) model with repeated measures was applied. Significant differences between pairs were determined using the studentized maximum modulus multiple comparison adjustment method, also known as Hochberg’s GT2 [33], which is utilized to evaluate significant differences between group means in the context of multiple pairwise comparisons. To address the violation of the normality assumption of ANOVA, the variables that exhibited a non-normal distribution were corrected by applying a monotonically ranked transformation. If the distribution remained non-normal after the transformation, a Friedman test was performed, a post-hoc analysis was carried out using Wilcoxon signed-rank tests, and a Bonferroni correction was applied. A significance level of $p<0.05$ was considered statistically significant.

## Results

All the parameters followed a normal distribution except for hip angle peaks, CoM in both directions, the first peak of vertical GRF, and the perceived exertion. Only the latter remained non-normal after the transformation. All the results of the statistical analysis are provided in the Additional file 1.

### Spatiotemporal, metabolic, and subjective parameters

**Fig. 2:**
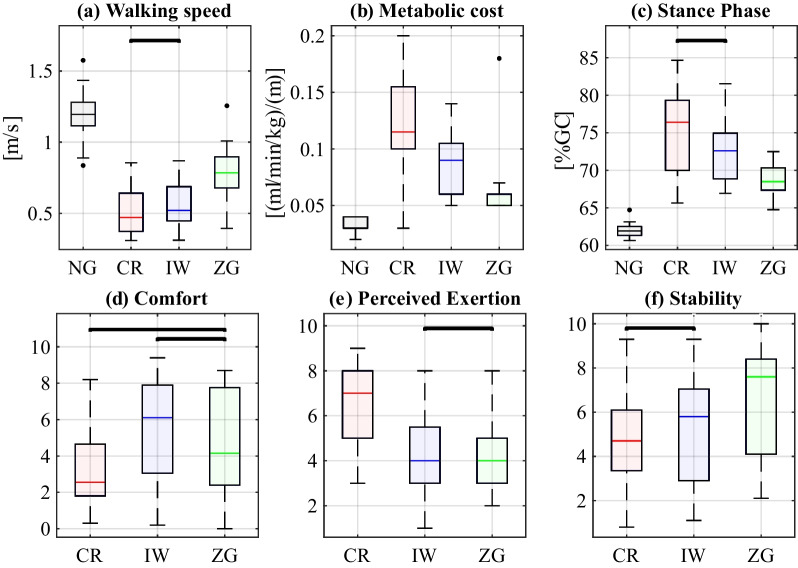
Summary statistics of the scalar parameters examined. The black horizontal lines indicate nonsignificant differences between paired conditions. **a** Walking speed during the 6MWT, **b** metabolic cost, **c** stance phase duration during the second-visit walking trials, **d** rated comfort, **e** rated perceived exertion, and **f** rated stability

The results of the average walking speed and the metabolic cost measured during the 6MWT are presented in Fig. 2a and b, respectively. All the devices caused a significant ($p<0.0001$) reduction in walking speed compared to NG (1.19 m/s). Among the devices, walking with the ZG (0.78 m/s) was significantly faster than walking with CR and IW (0.47 and 0.52 m/s, respectively). All the devices exhibited significantly greater metabolic cost than NG. Among the devices, ZG resulted in significantly lower metabolic cost than IW ($p=0.0006$) and CR ($p<0.0001$). The stance phase durations are shown in Fig. 2c. All devices resulted in significantly longer stance phase duration relative to NG (62%GC, $p<0.0001$), with ZG (68%GC) significantly shorter than CR (76%GC, $p=0.0005$) and IW (72%GC, $p=0.0011$).

The subjective participant ratings are presented in Fig. 2d–f. The perceived exertion using CR was significantly higher than both IW ($p=0.0004$) and ZG ($p<0.0001$), which showed similar ratings ($p<0.0001$). CR was also rated significantly less comfortable than IW ($p=0.002$), with nonsignificant differences between the other pairs. ZG was rated significantly more stable than IW ($p=0.017$) and CR ($p=0.042$), which showed nonsignificant differences.

### Joint kinematics

**Fig. 3:**
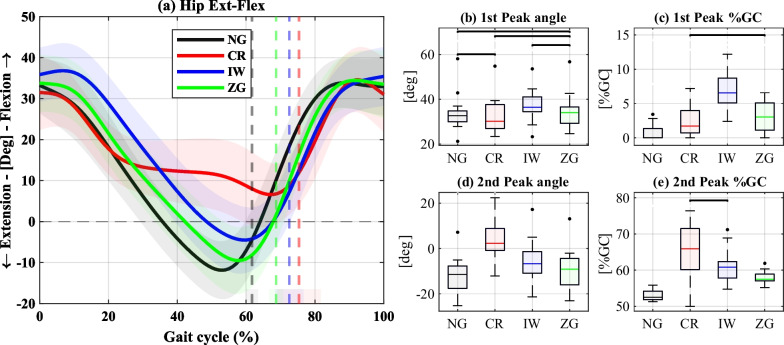
Sagittal plane hip angles of the weight-bearing leg. **a** Hip flexion-extension angles over a GC. The solid lines represent the medians, the shaded areas represent the range of all GCs, and the dashed vertical lines represent the mean of the TO events. **b–e** Summary statistics of the 1st peak of hip flexion angle **b** and timing **c**, and the 2nd peak of hip extension angles (**d**) and its timing (**e**). The black horizontal lines indicate nonsignificant differences

**Fig. 4:**
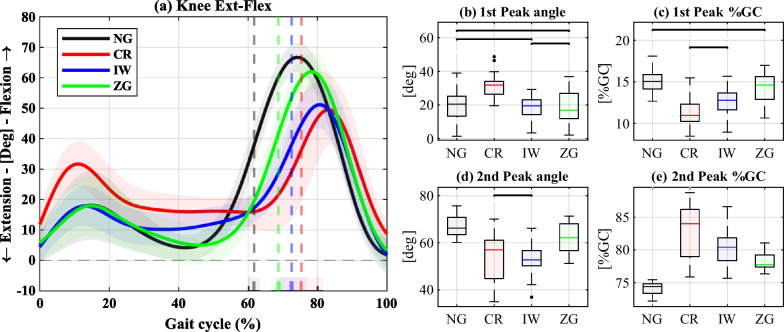
Sagittal plane knee angles of the weight-bearing leg. **a** Knee flexion-extension angles over a GC. The solid lines represent the medians, the shaded areas represent the range of all GCs, and the dashed vertical lines represent the mean of the TO events. **b–e** Summary statistics of the 1st knee flexion peak (**b**) and its timing (**c**), and the 2nd knee flexion peak (**d**) and its timing (**e**). The black horizontal lines indicate nonsignificant differences

**Fig. 5:**
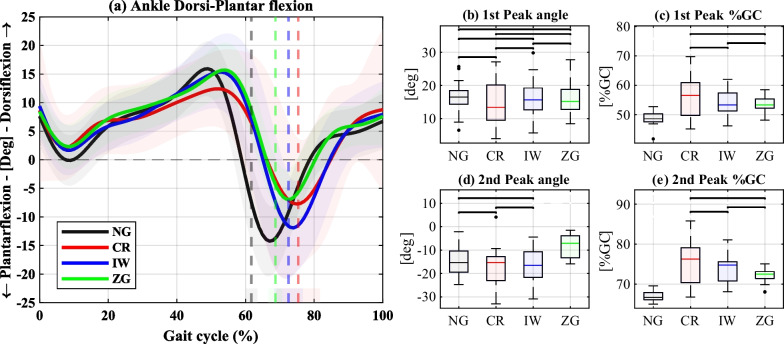
Sagittal plane ankle angle of the weight-bearing leg. **a** Ankle dorsiflexion-plantarflexion angles over a GC. The solid lines represent the medians, the shaded areas represent the range of all GCs, and the dashed vertical lines represent the mean of the TO events. **b**–**e** Summary statistics of the 1st ankle dorsiflexion peak (**b**) and its timing (**c**), and the 2nd ankle plantarflexion peak (**d**) and its timing (**e**). The black horizontal lines indicate nonsignificant differences

The results of the weight-bearing leg’s hip, knee, and ankle sagittal plane angles are shown in Figs. 3,  4, and  5, respectively. In each figure, panel (a) depicts the angles over a GC, panels (b) and (d) present selected peak values, and panels (c) and (e) the corresponding %GC in which they occurred. The full statistical results are provided in the Additional file 1.

Compared to NG, the first peak of the hip angle, corresponding to the maximum hip flexion at the beginning of the stance phase, was significantly higher for IW and nonsignificantly different for the other conditions. While this peak occurred right at IC for NG, all the devices significantly delayed its timing. The second peak, which typically corresponds to the maximum hip extension during late stance, was most significantly altered using CR, resulting in the absence of hip extension. Moreover, IW and ZG also caused a significant reduction and delay in hip extension, with the most extended delay obtained for CR, followed by IW and ZG.

The first peak of the knee angle, which corresponds to the maximum flexion during stance, exhibited a significant increase using CR compared to all other conditions. Conversely, using IW and ZG resulted in no significant differences from NG. The peak occurred significantly earlier using CR and IW, whereas ZG exhibited no significant difference relative to NG. The second peak, corresponding to the maximum knee flexion during swing, significantly decreased with all devices. However, ZG showed a significantly smaller reduction than CR and IW. All the devices resulted in significantly delayed timing relative to NG, with the longest delay obtained for CR, followed by IW and ZG.

The ankle angle first peak, corresponding to the maximum ankle dorsiflexion during late stance, showed no significant differences between the conditions. However, all the devices exhibited a delay in the peak, compared to NG. The second peak, corresponding to the maximum plantarflexion after push-off, significantly decreased using ZG and was significantly delayed by all the devices compared to NG.

### Center of mass

**Fig. 6:**
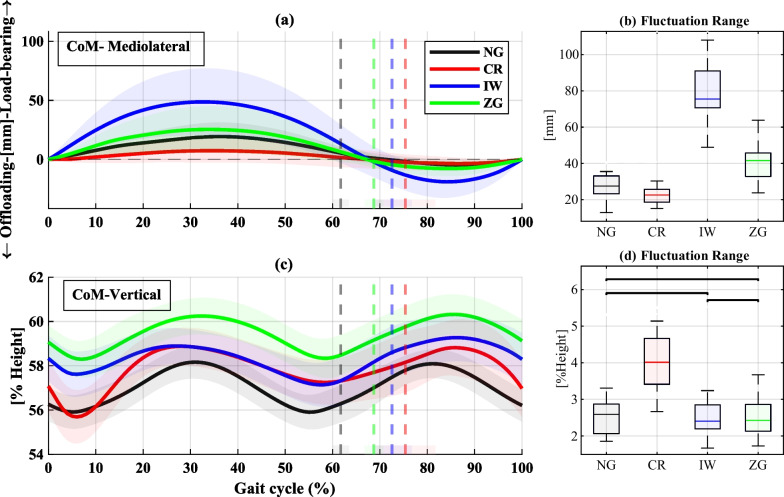
Mediolateral and vertical components of the CoM. **a** Mediolateral CoM trajectories over the GC, with positive values indicating the direction of the loadbearing leg. **b** Summary statistics of the range of fluctuation of the mediolateral CoM. **c** Vertical CoM trajectories over the CG, normalized by the participants’ height. **d** Summary statistics of the vertical CoM range of fluctuation. In **a** and **c**, the solid lines represent the median for each condition, the shaded domains represent the range of all cycles, and the dashed vertical lines represent the mean of the TO events. In **b** and **d**, black horizontal lines indicate nonsignificant differences

The mediolateral and vertical trajectories of the CoM are illustrated in Fig. 6. In the mediolateral direction, IW and CR exhibited significantly larger and lower CoM fluctuation ranges than all other conditions, respectively. The vertical CoM fluctuation range was similar for NG, IW, and ZG, whereas CR exhibited significantly larger fluctuations than all the other conditions.

### Ground reaction forces

**Fig. 7:**
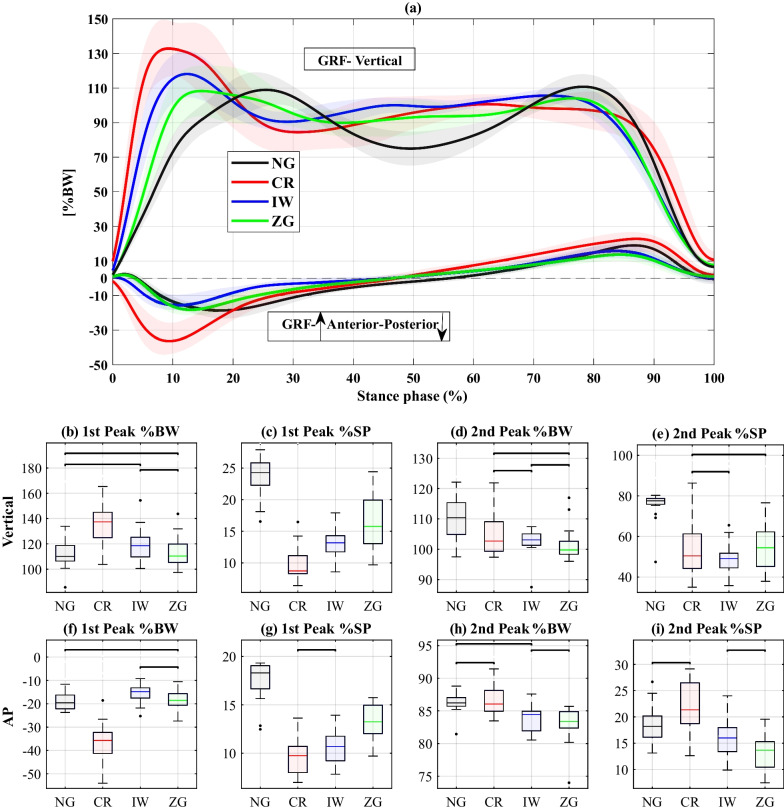
Vertical and anterior-posterior (AP) components of the GRF normalized by body weight (BW). **a** The trajectories of the vertical and AP trajectories over the stance phase. The solid lines represent the medians and the shaded areas represent the range of all cycles. **b**–**i** Summary statistics of the different GRF peaks: **b** 1st peak of vertical GRF, **c** timing of 1st vertical GRF, **d** 2nd peak of vertical GRF, **e** timing of the 2nd peak of vertical GRF, **f** 1st peak of AP GRF, **g** timing of 1st peak of AP GRF, **h** 2nd peak of AP GRF, **i** timing of  2nd peak of AP GRF. The black horizontal lines indicate nonsignificant differences

Figure 7 summarizes the results of the vertical and anterior-posterior GRFs of the weight-bearing leg over the stance phase. The first peak of the vertical GRF, occurring during weight acceptance, significantly increased using CR, compared to all other conditions. Moreover, it occurred significantly earlier using all devices than in NG, with the CR causing the most significant difference, followed by IW and ZG, the latter being closest to NG. The second peak of the vertical GRF, occurring during push-off, was significantly reduced using all the devices, with no significant differences among them. Moreover, for all the devices, the second peak occurred significantly earlier than in NG despite a larger variance caused by the flatter peaks. The magnitude of the first peak of the anterior-posterior GRF, corresponding to the braking force during weight acceptance, most significantly increased using CR and showed no significant difference between ZG and NG. This peak occurred significantly earlier using all devices, with the most significant difference for CR, followed by IW and ZG. The second peak, corresponding to the propulsion force during late stance, was less affected by the devices, although significant reductions in force and timing were exhibited for ZG.

### Pressure and pain feedback

The regions of pressure and pain reported by the participants are summarized in Table  1. The most frequently mentioned regions were the hands for CR and the shank for ZG and IW.

**Table 1:** The ratings of pressure and pain locations on the unloaded leg indicated by the participants for each device

Device	Rated pressure			Rated pain		
	Region	Number of participants	Intensity, mean ± SD	Region	Number of participants	Intensity, mean ± SD
CR	Hands	13	5.08 ± 1.87	Hands	4	5.94 ± 3.06
	Forearm	3	3.13 ± 1.90	Forearm	1	2.90
IW	Thigh	2	1.85 ± 1.35	Thigh	2	3.25 ± 2.55
	Shank	7	3.14 ± 2.46	Shank	3	2.80 ± 2.33
ZG	Shank	19	5.43 ± 1.99	Shank	12	5.52 ± 2.51
	Foot	6	3.80 ± 2.80	Foot	6	4.15 ± 2.29

## Discussion

This study examined the effects of three different devices for unilateral foot/ankle unloading on biomechanical, physiological, and subjective parameters measured during walking. Several studies have previously examined the effects of axillary or forearm crutches and hands-free knee crutch, such as IW. However, to the best of our knowledge, this is the first study to conduct a broad scope of comprehensive biomechanical analysis, metabolic cost, and subjective evaluation of an unloading AFO compared to other devices. Overall, the ZG AFO showed favorable results across most parameters but performed poorly in terms of comfort.

### Spatiotemporal, metabolic, and subjective parameters

Among the devices, the self-selected walking speed was significantly higher using ZG, but all the devices exhibited significantly slower walking speed than NG (Fig. 2a). Similarly, previous studies reported significantly slower walking using IW compared to NG [24, 34] and significantly faster walking with IW compared to CR [35]. Contrary to our findings, other studies found that participants walked slower with IW than with CR. However, they used axillary crutches [23, 34]. We selected forearm crutches based on their overall superior performance over axillary crutches reported in terms of walking speed, metabolic cost, and pressure on the upper extremities [5]. Since the post-hoc results showed significant differences in self-selected walking speed between the conditions, we conducted an additional statistical analysis with walking speed as a covariate variable, to evaluate the effect of walking speed on the other variables. The results of this analysis are included in the Additional file 1. Nevertheless, it is crucial to recognize that patients will naturally adopt a self-selected walking speed in real-life clinical scenarios. Therefore, evaluating parameter values without controlling for walking speed offers insights into the loads and motion that patients genuinely experience and provides a relevant and practical perspective.

We found the highest metabolic cost while using CR, followed by IW, ZG, and NG, with statistically significant differences between all pairs (Fig. 2b). The CR and IW results are consistent with previous research [34, 35]. Moreover, these results correspond well with the participants’ rated perceived exertion, which was significantly higher for CR, albeit comparable between IW and ZG. These differences in perceived exertion ratings between CR and IW are consistent with previous studies [23, 34]. The higher walking speed and lower metabolic cost of ZG support our hypothesis that the ZG would lead to a more natural gait pattern, resulting in a faster and more energetically efficient gait.

The significantly longer stance phase durations of the weight-bearing leg, observed using IW and CR (Fig. 2c) are consistent with the difference in walking speed [36], and with previous research [5, 24]. The participants may have increased the stance duration of their weight-bearing leg to compensate for their lack of stability, as indicated by their stability ratings. The ZG exhibited significantly higher stability rating and shorter stance phase than the other devices. Using CR and IW, participants shortened the swing phase of the weight-bearing leg, subsequently shortening the duration spent on the IW in single support and on the CR with no leg-ground contact, which are unstable configurations.

### Joint kinematics

All the devices altered the sagittal plane joint kinematics of the weight-bearing leg compared to NG. However, the ZG resulted in a walking pattern that was overall more similar to NG in most parameters. Particularly, hip extension (Fig. 3) at push-off was significantly reduced using IW (in agreement with previous research [24]), contributing to a shorter stride length and reduced walking speed. This is likely attributed to the challenge of swinging forward the leg fitted with the IW, given its relatively heavy mass and the limitation to knee flexion, making ground clearance a more challenging task. Using CR, the absence of hip extension was likely due to the forward inclination of the upper body, assisted by the CR [8]. The hip angles using ZG were most similar to NG, and the significant differences observed in the peak angles became nonsignificant once accounting for walking speed as a covariate (see Additional file 1). This suggests that the enabled knee mobility on the affected side contributed to a more natural walking pattern on the weight-bearing side. The significant delay in hip extension observed for all the devices is consistent with their longer stance phase.

Similarly, the knee flexion angles of the weight-bearing leg were less affected by ZG than CR and IW (Fig. 4). Particularly, CR caused a significantly larger knee flexion peak during stance, in agreement with previously reported results [8], whereas IW and ZG did not alter this peak significantly. However, after accounting for walking speed, the differences between NG-IW and NG-ZG became significant (see Additional file 1), which is consistent with previous findings associating slower walking speed with reduced peak knee flexion during stance [37, 38]. The second knee flexion peak, occurring during swing, was significantly lower using CR and IW (in agreement with previous findings [24]), consistently with the shorter swing period and instability reported with these devices. The use of ZG also reduced the peak of swing knee flexion, but significantly less than the other devices. As with the hip angle, the delay observed in the second peak is consistent with the delayed TO using the devices.

The effects of the devices on the weight-bearing ankle angles were less pronounced than the other joints (Fig. 5). The dorsiflexion angle during stance was nonsignificantly altered by all devices, and its delay was mainly due to the extended stance phase. Conversely, the push-off plantarflexion was significantly reduced by the ZG, but not by the other devices. However, this variable exhibited large variability, and when accounting for walking speed as a covariate, this significance reversed. The reduced push-off plantarflexion could be related to the shoe leveler worn on the weight-bearing leg during the ZG condition for equating the leg lengths. This may have caused the participants to hesitate to fully plantarflex their ankle, since the shoe leveler can slightly slip relative to the shoe. This difference can also explain the reduced push-off GRF using the ZeroG. Additionally, the ZG AFO has a locked ankle joint and a relatively long and flat sole, which might impair the initial roll-over motion of the affected leg occurring in parallel to the weight-bearing leg’s plantarflexion peak. Conversely, the IW has a short and rounded contact with the ground, which may have assisted in obtaining a more natural contralateral ankle push-off movement.

### Center of mass

Several significant differences have been identified in the patterns of the CoM (Fig. 6). The increased mediolateral CoM fluctuation observed with the IW may have resulted from the inability to flex the knee using IW, which required the participants to abduct their hips during swing (circumduction) to achieve proper ground clearance. This movement, together with the relatively large weight of the IW, required shifting of the CoM towards the unaffected leg, as evident in 6). In contrast, CR exhibited the smallest mediolateral CoM fluctuation, which indicates that the participants used the CR’s contact with the ground to propel their body forward in a straighter line. Although smaller mediolateral COM fluctuations may be attributed to improved balance, the participants rated CR as the most unstable.

Regarding vertical CoM, CR resulted in a significantly larger fluctuation range than ZG and IW, which exhibited fluctuations similar to NG. Note that the absolute values of IW and ZG are higher. For IW, this could be attributed to the lack of knee flexion, and for ZG this is a result of the added height of the device and the shoe leveler. Nevertheless, despite the higher CoMs, their fluctuation ranges remained similar to NG. Minimizing CoM vertical fluctuation is commonly thought to be related to minimized mechanical work and metabolic cost [39, 40], supporting our findings. However, it is noted that the opposite hypothesis also prevails, but it refers to able-bodied gait [41].

### Ground reaction forces

Several notable effects on the GRF patterns have been observed (Fig. 7). CR resulted in significantly higher braking GRFs in both vertical and posterior directions, consistent with previous findings [12, 19]. This can be explained by the weight-bearing foot contacting the ground after a short swing-through phase whereby the body accelerates forward, supported only by the crutches. The abrupt brake of this acceleration likely led to the elevated GRF values and rates of change (slope) seen for CR. These elevated peak forces and loading rates are even more prominent, considering that the walking speed was slower than in NG, for all devices. Since increased walking speed is associated with increased GRFs [37, 42], these differences would likely increase if compared at the same walking speed. This assumption is also supported by the statistical analysis that includes walking speed as a covariate variable (see supplementary file S1). Increased braking forces might be detrimental to the weight-bearing leg, particularly for patients with comorbidities. In contrast, the lower GRF braking peaks obtained using ZG and IW may be beneficial in limiting the risk of injury to the weight-bearing leg. The significant reduction in propulsive GRFs during push-off (second peak) for ZG and IW could also be explained by the slower walking speed, as also indicated by the nonsignificant differences from NG, when walking speed is taken as a covariate (see Additional file 1).

### Summary and participant feedback

Overall, if we consider a smaller deviation from natural unassisted gait a positive indicator, the ZG performed favorably in most metrics and could be viewed as a preferable alternative to CR and IW. However, the pressure and pain feedback provided by the participants reveals that it inflicted the most excessive pressure and pain, particularly on the shank region where the brace is tightened. This suggests that the soft calf lacer of the ZG may be inadequate for complete unloading, whereby the entire GRF is transferred through the shank. Instead, a rigid brace, similar to an open transtibial prosthetic socket, may provide improved results [43]. However, a rigid brace must be custom-made and not prefabricated. Furthermore, keeping the forefoot from contacting the AFO sole during late stance was challenging, in agreement with previously reported for patellar tendon bearing braces and casts [30, 44]. To avoid any contact between the forefoot and the AFO base, we had to increase the height of the heel above the AFO base and support the forefoot with a strap, which contributed to the discomfort reported by a few participants. The CR and IW caused discomfort to fewer participants, mainly on the hands and shank, respectively, aligning with previous reports [5, 23, 35]. Moreover, it is important to note that AFOs such as the ZeroG require a significantly longer time, usually a few minutes, to be put on. Therefore, in situations where quick assistance is needed for a short period of time, crutches may still be the preferable option.

### Limitations

This study encompasses several limitations. First, our study population was exclusively comprised of young, healthy individuals. While the fact that the participants did not have an injured foot may not significantly impact the results, given that the foot was completely unloaded during walking, it restricts the generalizability of findings to broader populations. Moreover, it is worth noting that this design allowed for the comparison of each parameter to the participant’s baseline. Future research should explore the effects of these devices on older individuals and patients with diverse injuries and pathologies. Second, we studied only walking at self-selected speed on level ground, whereas a rehabilitation process typically includes other activities of daily living, such as walking on uneven and inclined surfaces, stair ascent and descent, sit-to-stand, and more. Furthermore, additional biomechanical parameters, such as joint kinematics and kinetics in the transverse and coronal planes, and plantar pressure, should also be examined.

### Impact

Using assistive devices in situations that require unloading can provide valuable benefits across diverse domains, such as enhancing mobility, supporting independence, facilitating active participation in daily life, encouraging physical activity, and enhancing cardiovascular and metabolic health [42]. Choosing the right device plays a key role in maintaining functionality and mitigating adverse effects on the affected leg (e.g., muscle atrophy and bone density reduction in the proximal leg regions that can be mobilized and loaded), as well as the weight-bearing leg and upper body (e.g., nerve compression and fractures). Additionally, maintaining a more even weight distribution and natural gait pattern may lead to a shorter acclimation period with the device and enhanced safety and balance, although this still needs to be verified in future clinical studies.

Achieving consistent and proper adherence to offloading devices remains a challenge, particularly in diabetic foot ulcers [45]. To optimize their impact, it is crucial to understand how these devices affect biomechanics, energy consumption, and user experience. Informing healthcare professionals about the different multi-factorial effects of each device can help them choose the best device for a particular patient. Moreover, the insights gained from this study can lead to advancements in device design, overcoming the identified limitations, and resulting in improved user satisfaction and clinical effectiveness, thereby maximizing their impact in real-world healthcare scenarios.

## Conclusion

In summary, this study aimed to investigate the effect of three different devices for foot-ankle unloading on walking biomechanics, metabolic cost, and preference. Significant differences among the devices were identified across all parameters, with results from crutches and iWalk aligning with previous studies. The ZeroG demonstrated favorable performance in most aspects, highlighting the potential of AFOs in enhancing gait rehabilitation when unloading is necessary. However, ZeroG’s shortcomings in terms of comfort and sound-side ankle kinematics were evident.

These findings may offer valuable insights for researchers and clinicians, which could aid in informed decision-making regarding the prescription of such devices for patients with foot-ankle injuries and pathologies. Furthermore, future work may leverage these results toward the design of enhanced ankle-foot unloading devices that improve rehabilitation and patient care.

### Supplementary Information

**Additional file 1.** This file contains the statistical analysis results for all the investigated parameters. The left columns present the original analysis, and the right columns present the modified analysis, in which walking speed is a covariate variable. Results (p-values) that changed from statistically significant to nonsignificant in the modified analysis are highlighted in blue, and results that changed from nonsignificant to significant are highlighted in green.

## Data Availability

The datasets used and/or analysed during the current study are available from the corresponding author on reasonable request.

## Abbreviations

6MWT: 6 Minute Walk Test
AFO: Ankle foot orthosis
BW: Body weight
CoM: Center of mass
CR: Crutches
IC: Initial contact
IW: iWalk
NG: Normal gait
SP: Stance phase
TO: Toe off
ZG: ZeroG
