# Supplementary material for: Walking with unilateral ankle-foot unloading: a comparative biomechanical analysis of three assistive devices
Source: J Neuroeng Rehabil. 2024 Apr 30;21:67. doi: 10.1186/s12984-024-01333-w (PMC11059772; doi:10.1186/s12984-024-01333-w)
Supplement: Supplementary file 1 — Additional file 1. This file contains the statistical analysis results for all the investigated parameters. The left columns present the original analysis, and the right columns present the modified analysis, in which walking speed is a covariate variable. Results (p-values) that changed from statistically significant to nonsignificant in the modified analysis are highlighted in blue, and results that changed from nonsignificant to significant are highlighted in green. [file 12984_2024_1333_MOESM1_ESM.pdf]

		Original Analysis						Modified Analysis with walking speed as covariate variable					
	**Pairs**	**NG-CR**	**NG-IW**	**NG-ZG**	**CR-IW**	**CR-ZG**	**IW-ZG**	**NG-CR**	**NG-IW**	**NG-ZG**	**CR-IW**	**CR-ZG**	**IW-ZG**
**General Gait Variables**													
**Walking speed [m/s]**	**F value**	F (3,19) =104.85, p=<.0001											
	**t value**	-16.19	-15.24	-7.67	-1.35	6.19	7.13						
	**p value**	<.0001	<.0001	<.0001	0.6902	<.0001	<.0001						
**Metabolic cost [(ml/min/kg)/m]**	**F value**	F (3,19) =51.03, p=<.0001											
	**t value**	-9.56	-9.38	-4.28	5.01	6.65	-4.09						
	**p value**	<.0001	<.0001	0.0004	<.0001	<.0001	0.0006						
**Stance phase [%GC]**	**F value**	F (3,19) =74.32, p=<.0001						F (3,19) =40.21, p=<.0001					
	**t value**	9.5	11.34	-13.67	2.75	4.95	4.61	9.09	7.65	-8.97	3.98	5.54	3.61
	**p value**	<.0001	<.0001	<.0001	0.0704	0.0005	0.0011	<.0001	<.0001	<.0001	0.0008	<.0001	0.0019
**Joint Angles**													
**Hip_ 1stPeakAngle [deg]**	**F value**	F (3,19) =6.46, p=0.0034						F (3,19) =4.35, p=.0171					
	**t value**	-0.72	3.9	-1.01	-3.52	-1.3	2.78	-0.28	2.23	-1	-3.55	-1.44	2.47
	**p value**	0.9738	0.0056	0.8875	0.0133	0.7264	0.0666	0.9998	0.1947	0.8922	0.0123	0.6341	0.1232
**Hip_ 1stPeakAngle %GC**	**F value**	F (3,19) =55.43, p=<.0001						F (3,19) =32.97, p=<.0001					
	**t value**	3.66	12.24	-5.3	-6.53	-1.26	4.79	4.55	9.67	-5.54	-6.94	-1.14	5.33
	**p value**	0.0097	<.0001	0.0002	<.0001	0.7492	0.0008	0.0013	<.0001	0.0001	<.0001	0.8233	0.0002
**Hip_ 2ndPeakAngle [deg]**	**F value**	F (3,19) =64.42, p=<.0001						F (3,19) =47.93, p=<.0001					
	**t value**	11.83	4.72	-3.81	6.89	9.4	3.84	8.06	0.05	1.78	9.45	11.68	1.75
	**p value**	<.0001	0.0009	0.007	<.0001	<.0001	0.0065	<.0001	1	0.4106	<.0001	<.0001	0.43
**Hip_ 2ndPeakAngle %GC**	**F value**	F (3,19) =60.05, p=<.0001						F (3,19) =17.77, p=<.0001					
	**t value**	8.83	10.97	-9	1.5	4.72	4.39	6.07	5.11	-5.42	2.71	4.69	2.91
	**p value**	<.0001	<.0001	<.0001	0.5923	0.0009	0.0018	<.0001	0.0004	0.0002	0.0771	0.0009	0.0505
**Knee_ 1stPeakAngle [deg]**	**F value**	F (3,19) =31.15, p=<.0001						F (3,19) =41.91, p=<.0001					
	**t value**	7.37	0.06	0.2	9.33	7.47	0.21	9.03	3.06	-2.92	7.39	8.59	1.99
	**p value**	<.0001	1	1	<.0001	<.0001	1	<.0001	0.0363	0.0491	<.0001	<.0001	0.2931
**Knee_ 1stPeakAngle %GC**	**F value**	F (3,19) =55.31, p=<.0001						F (3,19) =26.04, p=<.0001					
	**t value**	-9.44	-6.95	2.12	-2.69	-7.22	-5.04	-7.15	-4.03	1.7	-2.47	-7.16	-4.45
	**p value**	<.0001	<.0001	0.2355	0.0802	<.0001	0.0004	<.0001	0.0042	0.4583	0.123	<.0001	0.0016
**Knee_ 2ndPeakAngle [deg]**	**F value**	F (3,19) =41.53, p=<.0001						F (3,19) =13.62, p=<.0001					
	**t value**	-5.6	-10.74	5.42	0.32	-3.76	-9	-3.73	-2.93	0.5	-0.71	-4.45	-5.05
	**p value**	0.0001	<.0001	0.0002	0.9997	0.0077	<.0001	0.0082	0.0487	0.9962	0.9765	0.0016	0.0004

**Knee_ 2ndPeakAngle %GC**	**F value**	F (3,19) =78.96, p=<.0001						F (3,19) =30.56, p=<.0001					
	**t value**	10.16	11.5	-13.2	3.76	5.73	4.33	8.91	5.59	-6.12	5.11	6.38	2.77
	**p value**	<.0001	<.0001	<.0001	0.0078	<.0001	0.0021	<.0001	0.0001	<.0001	0.0004	<.0001	0.0668
**Ankle_ 1stPeakAngle [deg]**	**F value**	F (3,19) =1.37, p=0.2826						F (3,19) =2.88, p=.0631					
	**t value**	-1.68	-0.09	0.58	-1.52	-1.17	0.22	-2.7	-1.79	2.22	-0.54	-1.4	-0.76
	**p value**	0.468	1	0.9915	0.5795	0.8029	1	0.0781	0.4027	0.1993	0.994	0.6552	0.9679
**Ankle_ 1stPeakAngle %GC**	**F value**	F (3,19) =31.78, p=<.0001						F (3,19) =12.88, p=<.0001					
	**t value**	4.86	5.21	-9.63	2.37	1.99	0.33	2.77	-0.85	-1.38	4.92	2.39	-2.32
	**p value**	0.0006	0.0003	<.0001	0.1485	0.2958	0.9996	0.0675	0.9462	0.673	0.0006	0.1438	0.1638
**Ankle_ 2ndPeakAngle [deg]**	**F value**	F (3,19) =38.90, p=<.0001						F (3,19) =44.23, p=<.0001					
	**t value**	-1.79	-1.55	-8.25	-0.44	-6.25	-6.54	-4.05	-4	-1.89	0.79	-7.84	-7.88
	**p value**	0.4009	0.5599	<.0001	0.9979	<.0001	<.0001	0.004	0.0045	0.3477	0.9603	<.0001	<.0001
**Ankle_ 2ndPeakAngle %GC**	**F value**	F (3,19) =36.08, p=<.0001						F (3,19) =14.43, p=<.0001					
	**t value**	6.23	7.9	-9.87	1.77	2.73	2.4	4.97	2.75	-4.37	3.81	3.2	0.36
	**p value**	<.0001	<.0001	<.0001	0.4151	0.0732	0.1402	0.0005	0.0707	0.0019	0.0069	0.027	0.9994
**Center of Mass**													
**CoM_Mediolateral fluctuation [mm]**	**F value**	F (3,19) =298.81, p=<.0001						F (3,19) =99.99, p=<.0001					
	**t value**	-3.11	18.83	-8.76	-20.02	-10.1	13.28	-2.86	16.85	-6.09	-15.75	-7.51	13.73
	**p value**	0.0326	<.0001	<.0001	<.0001	<.0001	<.0001	0.0563	<.0001	<.0001	<.0001	<.0001	<.0001
**CoM_Vertical fluctuation [%Height]**	**F value**	F (3,19) =82.01, p=<.0001						F (3,19) =65.49, p=<.0001					
	**t value**	9.99	-0.23	-0.03	10.86	8.93	-0.29	14.01	6.5	-5.55	7.37	10.01	2.98
	**p value**	<.0001	1	1	<.0001	<.0001	0.9998	<.0001	<.0001	0.0001	<.0001	<.0001	0.0437
**Ground Reaction Forces**													
**AP_GRF_ 1stPeak %BW**	**F value**	F (3,19) =50.94, p=<.0001						F (3,19) =58.93, p=<.0001					
	**t value**	-8.95	3.3	-0.7	-12.09	-9.43	2.74	-13.1	-4.75	5.3	-10.97	-11.45	-0.97
	**p value**	<.0001	0.0217	0.9774	<.0001	<.0001	0.0711	<.0001	0.0008	0.0002	<.0001	<.0001	0.9032
**AP_GRF_ 1stPeak %SP**	**F value**	F (3,19) =77.35, p=<.0001						F (3,19) =51.67, p=<.0001					
	**t value**	-13.89	-12.55	11.14	-1.86	-6.55	-8.19	-11.07	-5.93	5.57	-4	-7.27	-4.24
	**p value**	<.0001	<.0001	<.0001	0.3636	<.0001	<.0001	<.0001	<.0001	0.0001	0.0045	<.0001	0.0026
**AP_GRF_ 2ndPeak %BW**	**F value**	F (3,19) =27.44, p=<.0001						F (3,19) =22.83, p=<.0001					
	**t value**	2.48	-2.03	6.06	6.24	7.61	2.9	5.75	2.76	1.64	3.87	8.01	5.79
	**p value**	0.1206	0.2768	<.0001	<.0001	<.0001	0.0514	<.0001	0.0686	0.4949	0.0061	<.0001	<.0001
**AP_GRF_ 2ndPeak %SP**	**F value**	F (3,19) =29.34, p=<.0001						F (3,19) =32.09, p=<.0001					
	**t value**	0.78	-4.75	7.54	4.46	4.24	1.18	-1.86	-7.12	8.44	6.11	4.2	-0.13
	**p value**	0.9616	0.0008	<.0001	0.0016	0.0026	0.7978	0.3618	<.0001	<.0001	<.0001	0.0028	1
**VerticalGRF_ 1stPeak %BW**	**F value**	F (3,19) =12.67, p=<.0001						F (3,19) =44.34, p=<.0001					
	**t value**	5.38	2.42	-0.37	3.8	5.91	2.03	11.26	9.55	-8.01	1.94	8.27	7.09

	**p value**	0.0002	0.1369	0.9992	0.0071	<.0001	0.2766	<.0001	<.0001	<.0001	0.3182	<.0001	<.0001
**VerticalGRF_ 1stPeak %SP**	**F value**	F (3,19) =97.81, p=<.0001						F (3,19) =60.31, p=<.0001					
	**t value**	-15.02	-13.35	9.05	-4.23	-5.67	-3.34	-12.8	-7.04	6.25	-5.54	-5.99	-2
	**p value**	<.0001	<.0001	<.0001	0.0027	0.0001	0.0199	<.0001	<.0001	<.0001	0.0001	<.0001	0.2909
**VerticalGRF_ 2ndPeak %BW**	**F value**	F (3,19) =9.74, p=.0004						F (3,19) =8.25, p=.001					
	**t value**	-3.41	-4.35	5.23	1.04	2.63	0.92	0.1	0.65	2.03	-0.59	2.67	3.72
	**p value**	0.0168	0.002	0.0003	0.8724	0.0896	0.9224	1	0.985	0.274	0.9909	0.0825	0.0084
**VerticalGRF_ 2ndPeak %SP**	**F value**	F (3,19) =78.59, p=<.0001						F (3,19) =9.26, p=0.0005					
	**t value**	-7.11	-14.23	8.42	1.69	-0.43	-3.06	-4.24	-4.72	4.64	0.66	-0.2	-1.1
	**p value**	<.0001	<.0001	<.0001	0.4675	0.9983	0.0363	0.0026	0.0009	0.0011	0.9829	1	0.844
**Subjective rating**													
**Comfort**	**F value**	-	-	-	F (2,19) =8.14, p=.0028								
	**t value**	-	-	-	-4.03	-1.76	1.44						
	**p value**	-	-	-	0.0021	0.2476	0.4069						
**Perceived Exertion**	**χ2 value**	-	-	-	χ2 (2) =16.25, p=.0003								
	**S value**	-	-	-	71.5	74.5	-16.5						
	**p value**	-	-	-	0.0008	<.0001	0.399						
**Stability**	**F value**	-	-	-	F (2,19) =5.94, p=.0099								
	**t value**	-	-	-	-0.49	-2.69	-3.12						
	**p value**	-	-	-	0.9466	0.0417	0.0166						

Changed from significant to nonsignificant

Changed from nonsignificant to significant
